# Long COVID-19 in Children: From the Pathogenesis to the Biologically Plausible Roots of the Syndrome

**DOI:** 10.3390/biom12040556

**Published:** 2022-04-08

**Authors:** Michele Piazza, Maria Di Cicco, Luca Pecoraro, Michele Ghezzi, Diego Peroni, Pasquale Comberiati

**Affiliations:** 1 Department of Surgery, Dentistry, Pediatrics and Gynecology, University of Verona, 37126 Verona, Italy; michele.piazza@univr.it (M.P.); luca.pecoraro@univr.it (L.P.); 2 Department of Clinical and Experimental Medicine, Section of Pediatrics, University of Pisa, 56126 Pisa, Italy; maria.dicicco@unipi.it (M.D.C.); pasquale.comberiati@unipi.it (P.C.); 3 Allergology and Pneumology Unit, V. Buzzi Children’s Hospital, 20154 Milan, Italy; michele.ghezzi@asst-fbf-sacco.it

**Keywords:** COVID-19, SARS-CoV-2, long-COVID, vitamins, antioxidant, vitamin D, magnesium, selenium, zinc, oxidative stress

## Abstract

Long Coronavirus disease-19 (COVID-19) refers to the persistence of symptoms related to the infection with severe acute respiratory syndrome-coronavirus-2 (SARS-CoV-2). This condition is described as persistent and can manifest in various combinations of signs and symptoms, such as fatigue, headache, dyspnea, depression, cognitive impairment, and altered perception of smells and tastes. Long COVID-19 may be due to long-term damage to different organs—such as lung, brain, kidney, and heart—caused by persisting viral-induced inflammation, immune dysregulation, autoimmunity, diffuse endothelial damage, and micro thrombosis. In this review, we discuss the potential and biologically plausible role of some vitamins, essential elements, and functional foods based on the hypothesis that an individual’s dietary status may play an important adjunctive role in protective immunity against COVID-19 and possibly against its long-term consequences.

## 1. Introduction

Long Coronavirus disease-19 (COVID-19)—and the cognate term “long-haulers”—refers to the persistence of symptoms related to the infection with severe acute respiratory syndrome-coronavirus-2 (SARS-CoV-2) [1]. This condition is described as persistent and can manifest in various combinations of signs and symptoms in 10% [2] to 87% [3] of adults, particularly females, following SARS-CoV-2 infection [4]. More than 50 long-term effects of SARS-Cov-2 infection have been identified, including fatigue, headache, dyspnea, cognitive impairment—numbness, depression, altered perception of smells and tastes, poor appetite, chronic cough, joint and chest pain, postural orthostatic tachycardia expression of autonomic dysregulation, thermoregulation abnormalities, skin eruptions, and gastrointestinal disorders [5]. Similar findings have also been reported in children and adolescents [6]. Recent evidence on vaccination against SARS-Cov-2 suggests that vaccines reduce the risk of long COVID by lowering the chances of contracting COVID-19 in the first place. However, for those who do experience a breakthrough infection, the vaccination might only halve the risk of long COVID—or have no effect on it at all [7]. Understanding the prevalence of long COVID among vaccinated people has urgent public-health implications as restrictions that limited viral spread are eased in some countries. It could also offer clues about what causes lingering COVID-19 symptoms long after the acute infection has cleared [7].

Although long COVID-19 has been recently recognized as the next public health threat [8], the risk of persistent symptoms following SARS-CoV-2 infection is debated in the pediatric population, partly because of confusion around the definition [6]. Recently, using a protocol called the Delphi method, the WHO arrived at the definition of post-COVID-19 as “a condition which occurs in individuals with a history of probable or confirmed SARS-CoV-2 infection, usually 3 months from the onset of COVID-19 with symptoms that last for at least 2 months that cannot be explained by an alternative diagnosis” [9]. However, the WHO adds to their guidance that “a separate definition may be applicable for children” [9]. Similarly, the National Institute for Health and Care Excellence (NICE) suggests the definition of post-COVID-19 syndrome for people who still have symptoms for more than 12 weeks after the start of acute symptoms, but again no distinction was made between adults and children [10].

Despite all these limitations, in a recent review of 39 studies on 10,951 people in 12 countries, over 60 physical and psychological signs and symptoms were reported to persist up to 7 months after COVID-19 onset [11]

Such a complex and variable clinical picture can be difficult to interpret. The lack of specific diagnostic tests and the presence of cognitive bias [12] associated with a defect in the active listening of patients [13] can further lead physicians to disregard a biological basis for this multifaceted symptomatology [14].

Long COVID-19 may be due to long-term damage to different organs—such as lung, brain, kidney, and heart—caused by persisting viral-induced inflammation, immune dysregulation, autoimmunity, diffuse endothelial damage, and micro thrombosis [15]. Intestinal dysbiosis could also play an important pathogenetic role given the close connections between the intestine immune system and central nervous system [16]. Furthermore, reactivation of the Epstein-Barr virus (EBV) has been documented in more than half of long haulers [17]. This is not a trivial effect because EBV reactivation has been associated with skin, cardiovascular, hematological, and neurological complications [18], which may also occur in long COVID-19.

Considering these pathogenic mechanisms, it seems essential to investigate the possible and hypothetically preventable deep roots of this new pandemic. In this review, we discuss the potential and biologically plausible role of some vitamins, essential elements, and functional foods [19] based on the hypothesis that an individual’s dietary status may play an important adjunctive role in protective immunity against COVID-19 [20] and possibly against its long-term consequences. The novelty of this short review article is to attract the attention of researchers to the possible predisposing factors to the development of Long COVID-19 and thus on the possibility to attenuate its prevalence acting on epigenetic factors, as observed in ecological studies [21,22,23,24].

## 2. Methods

A literature search was performed in September 2021 across MEDLINE/PUBMED to identify studies investigating the possible role of nutraceuticals in long COVID-19. Original studies were selected in priority, followed by systematic reviews and meta-analyses. We included randomized controlled trials, observational (cross-sectional and cohort), and case-control studies, both in children and adults, which were peer-reviewed and written in English. The following search terms were used: *SARS-CoV-2* or *COVID-19* or *long COVID* and *persistence of the SARS-CoV-2 virus* and *spike protein and *biologic therapies* and *nutraceutical* and *supplements* and *therapy*. The special interest was in studies published within the previous 24 months.

## 3. The Possible Deep Roots of Long COVID-19 and Their Biological Plausibility

Besides genetic predisposition, a diet poor in anti-inflammatory/antioxidant substances with potential immune-modulating and anti-viral activity can be a predisposing but preventable factor for more severe SARS-Cov-2 and very likely also for the development of persistent long symptoms after the acute phase of the disease. This proposition emerged from epidemiologic studies demonstrating that populations with very low death rates were found to have an unusual common feature of eating large quantities of fermented vegetables, including members of the cruciferous and Brassicaceae family [25]. At the beginning of the pandemic, it was suggested that treatment with a wide range of existing host-directed therapies, including nutrient supplements, was possibly beneficial in the care of 2019-nCoV infection [26]. Vitamin D deficiency has been associated with an increased number of cases with severity, and with deaths [27]. In COVID-19 infection, zinc deficiency was found to be linked to higher odds of complications, including deaths [28], particularly when combined with selenium deficiency [29], and selenium supplementation has been associated with a better prognosis in those patients [30]. In addition, low levels of magnesium, which are usually present in all COVID-19 comorbidities [31], are associated with an increased inflammatory state [32], while an increased likelihood of survival is seen in severe patients with COVID-19 with higher magnesium blood level on admission to hospital (odds ratio for mortality of 0.032) [33]. Collectively, these studies suggest that nutritional support may effectively reduce inflammation and oxidative stress, thereby strengthening the immune system during the COVID-19 crisis, and ecological studies have lent support to this suggestion [25,34,35]. These supplementations may offer additional benefits, providing significant antiviral, anti-inflammatory, antithrombotic, and cytoprotective effects, thus preventing further tissue damage [36] and favorably modifying the gut microbiome which, in patients with COVID-19, has been found to be concordant with the disease severity and plasma concentrations of several inflammatory cytokines, chemokines, and blood markers of tissue damage, as well as the persistence of symptoms [37].

## 4. What Could Be Done for the Prevention

### 4.1. Vitamins

#### 4.1.1. Vitamin B Group

B group vitamins represent essential micronutrients for energy metabolism, DNA and protein synthesis, and immune cell regulation [38]. Vitamin B1 influences mitochondrial membrane potential, cytochrome C release, protein kinases, and p38-MAPK; suppresses oxidative stress induced by nuclear factor-kappa b (NF-κB); and has anti-inflammatory properties. Deficiency of vitamin B1 may cause dysfunction of the nervous system; neuroinflammation; T cell infiltration; chemokine CCL2 activation; overexpression of proinflammatory cytokines such as interleukin (IL)-1, tumor necrosis factor (TNF), IL-6, and arachidonic acid products; and induces expression of CD40 by the microglia and CD40L by astrocytes, which provoke the death of neurons [39]. The active form of vitamin B6, pyridoxal 5′-phosphate (PLP), has consistently been shown to be low in inflammatory conditions and inversely associated with numerous inflammatory markers in clinical and population-based studies, and its low concentration predicts the risk of chronic diseases [40]. Furthermore, PLP serves as a co-factor in neurotransmitter biosynthesis, as well as a scavenger of reactive oxygen species (ROS) [41]. Vitamin B12 appears to possess antioxidant properties by scavenging ROS, by the preservation of glutathione, modulation of cytokine and growth factor production and reduction of oxidative stress caused by advanced glycation end-products [42]. Furthermore, Vitamins B1 (thiamine), B6 (pyridoxine), B12 (cobalamin), and folate play an important role in the pathogenesis of neuropathy and neuropathic pain and on the inflammatory basis of depression [43]. Pyridoxal-5-phosphate and methylcobalamin are cofactors in peripheral nerve functions and cobalamin facilitates myelinogenesis and nerve regeneration [44]. Methyl-folate has been shown to improve endothelial function [45], and the vitamin B group is an essential nutrient for host gut microbiota [46]. In effect, supplementation of vitamin B12 together with vitamin D and magnesium was shown to prevent severe outcome progression in patients with SARS-Cov2 [47].

#### 4.1.2. Vitamin C

Vitamin C is one of the body’s most important antioxidants and is involved as a co-factor in the synthesis of carnitine; the formation of serotonin, dopamine, and nitric oxide; the synthesis of noradrenaline; the biosynthesis of amidated peptides; hypomethylation of DN; and the degradation of the transcription factor hypoxia-inducible factor 1 alpha implicated in energy metabolism [48]. Vitamin C contributes to immune defense by supporting various cellular functions of both the innate and adaptive immune system [48], with a possible preventive effect on autoimmune diseases [49]. Furthermore, fatigue, pain, cognitive disorders, and depression-like symptoms are known symptoms of vitamin C deficiency [50] and, although vitamin C plasma levels have not been evaluated in patients with long COVID-19, a deficit is most probable because infections are known to be coupled with high intakes of vitamin C, and insufficiencies in acute infections are frequent [48]. Indeed, a systematic review of studies evaluating the effect of this vitamin on low energy and weakness suggested that high dose intravenous vitamin C could be a beneficial treatment option in treating fatigue in patients with long COVID-19 [51].

#### 4.1.3. Vitamin D

**Figure 1:**
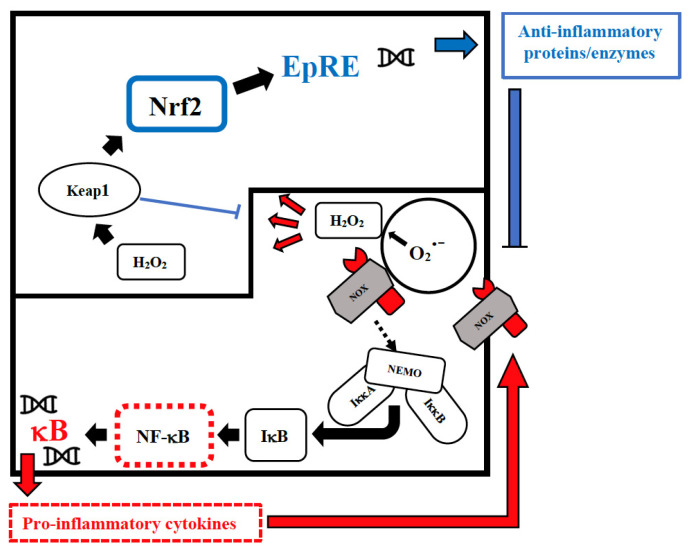
Intranuclear signal transductions can occur in two different pathways: while nuclear factor kappaB (NF-κB) tends to enhance and perpetuate the inflammatory response by triggering the expression of pro-inflammatory cytokines, nuclear factor erythroid 2–related factor 2 (Nrf2) activation through Kelch-like ECH-associated protein-1 (Keap1) oxidation dampens pro-inflammatory signaling by expression of peroxidases and other anti-inflammatory proteins. As E3-ligase, Keap1 also primes inhibitor of NF-κB kinase subunit beta (IKKβ) to degradation via ubiquitination, thereby directly interfering with NF-κB activation. For the sake of clarity, only the reactive oxygen species (ROS)-producing enzyme NADPH oxidase (NOX)-derived H2O2 is shown as an oxidant signal. Depending on the cellular system and the inflammatory stimulus, NOX-derived H_2_O_2_ may be supported or replaced by mitochondrial H_2_O_2_, lipoxygenase products, and S-alkylating electrophiles derived therefrom. NEMO, NF-κB essential modulator; IκB, Inhibitor of NF-κB.

Vitamin D has an immune-modulating effect and reduces the frequency of infections when taken in daily doses [52]. The discordant results related to its effect can be attributed to its erroneous use in cumulative monthly doses that are associated with a sudden increase in its plasma levels, followed within a few days by their drastic reduction due to the catabolic effect of the 24-hydroxylase that transforms the vitamin in inactive metabolites [53]. These continuous oscillations not only neutralize its effect but could also be dangerous in relation to its epigenetic action. Furthermore, this vitamin reduces cellular damage from oxidative stress and stimulates the Nrf2 pathway of signal transduction by promoting the synthesis of anti-inflammatory cytokines (Figure 1) [54].

In the specific case of COVID-19, numerous studies have shown that subjects deficient in vitamin D (<20 ng/mL) are twice as likely to test positive from a molecular swab [55], to be hospitalized in intensive care, and to have an inauspicious outcome of the disease [27]. In hospitalized patients, its use in a high daily or weekly dose significantly (−67%, −87%) reduced COVID-19 mortality [56], most likely also in relation to its T regulatory cells-boosting effect [57], and to its protective effect on alveolar damage, endothelial dysfunction [58], and alteration of heart rhythm [59]. Moreover, vitamin D has a preventive effect on the development of autoimmunity [60], including type 1 diabetes whose incidence increased during the pandemic, particularly in association with another autoimmune condition, celiac disease [61]. Moreover, this vitamin is an important regulator of the gastrointestinal microbiota [62], and its supplementation seems to mitigate EBV reactivation [63] and to provide help in depressed subjects with its deficiency [64]. In consideration of Vitamin D effects, it is biologically plausible to hypothesize its deficiency in subjects suffering from long symptoms after SARS-CoV-2 and to recommend its daily supplementation as an adjuvant treatment.

#### 4.1.4. Vitamin E

Vitamin E (alpha-tocopherol) is a fat-soluble vitamin and a potent antioxidant important in protecting cells from oxidative stress, regulating immune function [65], maintaining endothelial cell and heart integrity, and balancing coagulation and gut microbiota [66,67]. It has been demonstrated that vitamin E deficiency impairs the normal functions of the immune system in animals and humans, which can be corrected by vitamin E repletion [65]. Furthermore, a low level of vitamin E associated with selenium insufficiency results in specific viral mutations, changing relatively benign viruses into virulent ones [68]. In relation to the long COVID-19 problem, it was reported that a low level of serum alpha-tocopherol improved during the remission phase, as compared to the exacerbation phase, in patients with chronic fatigue syndrome, suggesting that increased oxidative stress may be involved in the pathogenesis and the severity of the symptoms of the syndrome [69].

### 4.2. Essential Elements

#### 4.2.1. Magnesium

Magnesium is the most abundant divalent cation in living cells and plays essential roles in the regulation of cell growth, division, and differentiation [70]. In the heart, magnesium plays a key role in modulating neuronal excitation, intracardiac conduction, and myocardial contraction by regulating several ion transporters, including potassium and calcium channels. Magnesium also has a role in regulating vascular tone, atherogenesis, and thrombosis, and proliferation and migration of endothelial and vascular smooth muscle cells [71], and it also acts protectively against phosphate-induced kidney injury [72]. It is involved in numerous biological processes (estimated at over 600) and, when present in physiological concentrations, it controls redox homeostasis, reducing the production of oxygen-derived free radicals in various tissues, lowering inflammation. Mechanisms include its “calcium-channel blocking” effects that lead to downstream suppression of NF-κB, IL-1β, IL-6, and TNF-α, as well as C-reactive protein (CRP) production [73]. Latent magnesium deficiency is associated with chronic low-grade inflammation [74], hypertension, metabolic syndrome, type 2 diabetes, and cardiovascular disease [75], as well as with increased levels of free radicals and mitochondrial dysfunction [76], possibly causally related to fatigue and myalgic encephalomyelitis/chronic fatigue syndrome [77], a common manifestation of long COVID-19. Furthermore, magnesium deficiency also increases platelet aggregation and adhesiveness and inhibits the growth and migration of endothelial cells, potentially altering microvascular functions [78], which is another important pathogenic effect in both SARS-Cov-2 and its long-term consequences. In addition, magnesium is necessary for the biosynthesis, transport, and activation of vitamin D [79], another key factor in the pathogenesis of immune dysfunction. A growing body of evidence supports the idea that magnesium supplementation prevents or treats various types of disorders or diseases related to the respiratory system, reproductive system, nervous system, digestive system, and cardiovascular system as well as kidney injury, and diabetes [80], which support the possibility of magnesium supplementation as a supportive treatment in patients with COVID-19 [81]. Co-supplementation of magnesium, selenium, and coenzyme Q10 in patients with benign thyroid conditions was associated with a significant drop in antibody titers and normalization of thyroid morphology [82], which should be further studied considering that subacute thyroiditis, autoimmune thyroiditis, and an atypical form of thyroiditis are complications of COVID-19 [83].

#### 4.2.2. Selenium

Selenium is an essential trace element for mammalian redox biology. Unlike other trace elements that act as cofactors, dietary selenium is converted in the body into aminoacid selenocysteine, which is then incorporated into one of the twenty-five selenoproteins [84] such as glutathione peroxidase, thioredoxin reductases, and methionine sulfoxide reductase, which are important components of the antioxidant defense systems. Reduced expression of selenoproteins as a result of low/sub-optimal selenium status could alter the molecular pathways involved in stress responses and contribute to an aggressive pro-inflammatory environment due to an imbalance between NF-κB and nuclear factor erythroid 2-related factor 2 (Nrf2) signaling (Figure 1), which may lead to poorer viral disease prognosis [85]. On the contrary, selenium supplementation is associated with lower expression of pro-inflammatory NF-κB signaling [86]. ROS are produced during viral infections, with both positive and negative consequences for the cell [87]. For example, phagocytic cells produce large amounts of ROS to eliminate a wide variety of pathogens without altering the host cell viability, but ROS have also been found to stimulate viral replication [88]. This fact is particularly significant for RNA viruses that exhibit the highest known mutation rates, with up to one mutation per genome per generation cycle [89], and selenium deficiency increases the pathogenicity and severity of infections by benign or mildly virulent strains of Coxsackie and influenza viruses, giving rise to multiple changes in the viral RNA [90]. Thus, dietary insufficiency of this oligo element impacts not only the immune response of the host, but also the virus itself, and dietary selenium deficiency, which causes oxidative stress in the host, can alter a viral genome so that a normally benign or mildly pathogenic virus becomes highly virulent in the deficient host. This has been shown in animal models for the influenza virus [91] and human coxsackie enterovirus [92]. Once the viral mutations occur, even hosts with a normal diet would be sensitive to the newly pathogenic strain [84]. A relation between selenium deficiency and human disease was first established by the finding in China that the etiology of Keshan disease implicated both coxsackievirus infection and a low intake of this micronutrient [93]. Keshan disease was characterized by blood selenium concentrations of <20 μg/L (0.25 μM) in patients who presented cardiac enlargement, congestive heart failure, and pulmonary edema. The disease mainly affected infants, children, and women of childbearing age and caused the death of thousands of people every year, but it disappeared when selenium fertilizer was applied to the soil to increase its content in the food chain and when people living in the endemic area were supplemented with selenium [93]. These findings reveal that the host nutritional condition and, above all, its antioxidant defense system can significantly contribute to the progression of benign viral genomes into more virulent viruses with increased transmissibility and pathogenicity.

Furthermore, many studies revealed that an association of selenium deficiency with thyroid autoimmunity selenium supplementation could significantly reduce thyroid autoantibodies in patients with Hashimoto’s thyroiditis [94], a possible long-term complication of SARS-Cov-2 [95], and its supplementation, particularly when associated with zinc has a beneficial effect on thyroid function [96] and in preserving immune tolerance [97]. Additionally, this oligo element exerts a protective effect on endothelial dysfunction [98], as well as on increased platelet-dependent thrombosis [99]. Finally, epidemiological studies have suggested an inverse association between selenium levels and inflammatory bowel disease. Changes in the cellular oxidative state coupled with altered expression of selenoproteins in macrophages drive the switch from a proinflammatory phenotype to an anti-inflammatory phenotype to efficiently resolve inflammation in the gut, restore epithelial barrier integrity [100], and establish an advantageous intestinal microbiome [101]. The possible effect of magnesium, selenium, and zinc in the promotion of mental health is currently under investigation [102].

#### 4.2.3. Zinc

Zinc is the second-most abundant trace metal in the human body after iron and an essential component of protein structure and function. It is a vital micronutrient for maintaining cellular physiology [103]. In fact, it is a structural component of ~750 zinc-finger transcription factors [104], allowing gene transcription, and it is a catalytic part of approximately 2000 enzymes on all sides of 6 classes (hydrolase, transferase, oxidoreductase, ligase, lyase, and isomerase) [9]. Zinc acts as a second messenger comparable to calcium [9,104]; thus, it is obvious that cellular signals are altered due to changed intracellular zinc concentrations. Therefore, zinc is biologically indispensable for cellular processes, including growth and development, as well as DNA synthesis and RNA transcription [103]. Additionally, zinc contributes to red-ox homeostasis because oxidative stress induces zinc release from metallothioneins as a mechanism to reduce ROS generated by mitochondrial dysfunction or viral infection [105]. Furthermore, zinc deficiency increases IL-6-induced activation of the JAK-STAT3 signaling pathways, which are normalized after zinc supplementation [106]. Zinc is known to be essential, especially for proper T cell and B cell development. During zinc deficiency, the recruitment of naïve Th cells and the percentage of cytotoxic T lymphocytes precursors is diminished, respectively [107]. Zinc inhibits NF-κB signal transduction with the consequent decreased expression of IL-1β and TNFα and decreased CRP levels, lipid peroxidation, and inflammatory cytokines and adhesion molecule expression [108]. More importantly, in the context of COVID-19 pathogenesis and its long-term consequences, zinc mediates the reduction of pro-inflammatory Th17 cells [109], and its deficiency increases endothelial dysfunction [110] and autoimmune susceptibility in general [96]. It has been repeatedly demonstrated that autoimmune diseases are associated with zinc deficiency [111], and an overreacting immune response can be beneficially influenced by the administration of zinc, which seems to be promising to improve the life of patients suffering from autoimmune diseases [112]. In addition, several studies have documented a positive association between zinc deficiency and the risk of depression, and an inverse association between zinc supplementation and depressive symptoms [102].

### 4.3. Phytochemicals: The Low Hanging Fruit

Viruses probably appeared as parasites of the first bacterial cells over 3.5 billion years ago [113], while plants and homo sapiens appeared on earth 450 million and 300,000 years ago, respectively [114]. Therefore, plants have hundreds of millions of years of greater experience in antiviral defenses than animals, and have most likely developed effective and non-specific defenses, i.e., valid against different viruses. Phytochemicals are naturally occurring plant chemicals that have been used in traditional medicines since ancient times and comprise various bioactive compounds that have now been classified as Alkaloids, Polyphenols, Carotenoids, and Organosulfurs, which are considered a natural weapon against inflammation and oxidation-mediated diseases [115]. Natural substances contained in fruit and vegetables, such as resveratrol, quercetin, sulforaphane, and curcumin, to name but a few, all have a stimulating effect on the intranuclear pathway of transduction of the Nrf2 signal and an inhibitory effect on the NF-κB pathway (Figure 1) [116], with the result of limiting the effect of the cytokine storm that occurs in patients with severe COVID-19 [117] and persistent inflammation and autoimmunity [118] that may occur in long COVID-19. These substances also exert an antiviral effect, both by binding to viruses outside the cell with electrostatic charges, and by preventing the binding process with their receptor, but also by limiting intracellular viral replication and hindering the escape of newly formed viruses from the cell [68]. In fact, the addition of resveratrol to cell cultures infected with the SARS-2 virus prevents its replication and the consequent cell damage [119] in relation to the conformational isomeric interaction between this polyphenol and the enzymes that all viruses use to replicate. The same inhibitory effect has been documented for quercetin [120] sulforaphane and curcumin [121]. The central structure of many synthetic antivirals consists of three rings present in most polyphenols [122]. Furthermore, a not negligible fact in relation to the pathogenetic phenomena of SARS-CoV-2 and its long-term prognosis is that almost all polyphenols and flavonoids also have antiarrhythmic [123], antiplatelet properties, and preventive effects on thrombotic events [124]. For example, the administration of quercetin in patients at risk of thrombosis for neoplastic diseases mitigates this danger [125] just as, in a population study, the consumption of apples, which are rich in polyphenols, reduces the risk of cerebrovascular disease [126]. Furthermore, these substances of plant origin attenuate the risk of pulmonary fibrosis in relation to their stimulating effect on the production of factors that inhibit leukocyte proteases [127]. Curcumin has been shown to be effective in reducing the symptoms of patients with COVID-19 by reducing the production of IL-1 and IL-6, halving the risk of death in subjects treated with the active ingredient, compared to those treated with placebo [128]. The pathogenetic mechanism underlying these results is represented by the ability of this plant extract to reduce the number and function of Th17 lymphocytes as well as the tendency to develop thrombosis [129], both of which are strongly involved in the pathogenesis of the disease and its long-term consequences. Furthermore, it has been shown that Curcumin lowers oxidative stress, inflammation, and pain, thus improving performance and post-exercise recovery [130] and has neuroprotective effects [131]. Sulforaphane has also been shown to be effective in three patients with COVID-19 of varying severity [132] in a clinical evaluation aimed at verifying the validity of the epidemiological observation that, in European countries, mortality from COVID-19 is reduced with increased consumption of cabbage, which is particularly rich in sulforaphane [25]. In addition to being a powerful stimulator of the Nrf2 signal, sulforaphane stimulates the synthesis of ATP at the mitochondrial level and promotes the metabolism of glucose and lipids [20]. Regarding SARS-CoV-2, an anonymous questionnaire answered by people who, during the first wave of the SARS-CoV-2 pandemic, were taking supplements containing many of the substances discussed above, highlighted how the swab for COVID-19 was negative in all 107 subjects who presented suggestive symptoms, and positive in only 33 out of 127 (25.9%) who had had close contact with sick persons [133]. At that time, the epidemiological data showed that, in the case of close contact, the percentages of positive subjects varied in a range that fluctuated from 42% [134] to 53% [30]. Among the subjects described in the study, no individual required hospitalization or emergency room checks. This observation supports the hypothesis put forward by North European authors who underline that optimal levels of trace elements, vitamins, and natural substances with antiviral, anti-inflammatory, antiplatelet, and antioxidant action can play both a preventive role against infections and a therapeutic adjuvant against viral diseases [135] and may protect against their complications.

## 5. Future Perspectives

In addition to the evaluation of comorbidities and immunological, inflammatory and coagulation parameters, a validated food frequency questionnaire should be used in children with long COVID-19 and in a comparator group [136]. Furthermore, appropriate evaluation of the concentration of the potentially protective elements listed in Table 1 should be considered in future studies to identify potentially epigenetic and thus potentially modifiable factors that underlie this new syndrome.

Preliminary evidence suggests that personalized rehabilitation with light and paced aerobic activity based on individual capacity may help some patients with long-term COVID-19 [15]. The use of drugs used in other similar conditions, such as myalgic encephalomyelitis, chronic fatigue syndrome, postural orthostatic tachycardia syndrome, and mast cell activation syndrome, is also being evaluated. However, at present, no drug is effective in ameliorating or alleviating the symptoms, radiological abnormalities, or inflammatory biomarkers of long COVID-19 [137].

For this reason, it seems implicit that the task of pediatricians, as well as of the general practitioner, is essentially to prevent, but also to evaluate the possibility of attenuating the symptoms with the use of the biologically plausible elements summarized in Table 1 [138]. A multi-disciplinary management is necessary, and the possible use of different nutraceuticals and essential elements, already suggested for COVID-19 patients [21,22,23,24] and in children with different inflammatory disease, seems reasonable [135,139,140].

Even though there is little data to suggest that the nutraceuticals and essential elements mentioned in this review are toxic when used in an appropriate dosage, interactions between nutraceuticals, especially when overdosed, and certain drugs leading to altered drug bioavailability should be carefully evaluated, and patients need to disclose supplement use to their physicians [141].

## 6. Conclusions

It is evident that some individuals, including those with mild initial symptoms of COVID-19, may suffer from variable and debilitating symptoms for many months after the initial infection. These long-term post-COVID-19 sequelae can be very debilitating, even in children, and lead to long absences from school. In consultation with parents and children, we pediatricians must acknowledge the limits of our knowledge; show interest in family experiences and the problems presented; and, at the same time, offer support, help, and reference. The fact that COVID-19 may have a long-term impact on children as well, including those with asymptomatic/paucisymptomatic COVID-19, highlights the need for pediatricians, mental health experts, and policymakers to take appropriate measures to reduce the impact of the pandemic on children’s health.

## Figures and Tables

**Table 1:** Vitamins, oligoelements, flavonoids, and polyphenols: their antiviral, anti-inflammatory, and antioxidant activities and their preventive effects against viral infections and their long-term consequences.

Componet	Mechanism of Action	References
Vitamin D	- immune modulating effect, reduces the frequency of respiratory infections	[51]
	- antioxidant and NrF2 stimulating effects thus increasing the synthesis of anti-inflammatory proteins	[51,142,143,144]
	- increases resistance to COVID-19 infection	[54]
	- reduces mortality related to COVID-19 complications	[27,55]
	- increases phagocytosis and killing of Streptococcus pneumoniae	[145]
	- reduces thrombosis risk	[146]
Vitamin E	- reduces oxidative stress	[147]
	- improves immune functions	[147]
Zinc	- anti-inflammatory effects inhibiting NF- κB activation	[148]
	- reduces oxidative stress induced damages on epithelial cells	[148]
	- improves immune defenses by:	[149]
	(1) increasing NK and cytotoxic CD8+ cell activity	[150]
	(2) reducing viral replication	[151]
	(3) reducing respiratory tract infections	[151]
	- suggested favorable effects on COVID-19:	[149]
	(1) improved cilia morphology and increased ciliary beat frequency	[149]
	(2) up-regulation of tight junction proteins ZO-1 and claudin-1with increased epithelial barrier effect	[149]
	(3) inhibition of RNA dependent polymerase—an essential enzyme for viral replication	[149]
	(4) up-regulation of IFNα production and up-regulation of antiviral proteins (RNaseL and PKR) known to degrade viral RNA and inhibit its translation	[149]
	(5) anti-inflammatory activity through inhibition of NF-κB signalin, g resulting in down-regulation of proinflammatory cytokine production	[149]
	(6) inhibition of S. pneumoniae growth through modulation of bacterial Mn (II) homeostasis	[149]
	- supplemented in daily doses of 7 mg in the elderly reduces pneumonia mortality by 39% and all-cause mortality by 53%	[152]
	- supplemented in adults reduces duration of symptoms of respiratory infections by 47%	[153]
	- its deficiency is associated with poor COVID-19 outcomes	[28,29]
Selenium	- in association with vitamin E, reduces ROS formation	[154]
	- promotes cell-mediated (TH1) immune response	[155,156,157]
	- enhances the function of cytotoxic effector cells, maintains T cell maturation and functions, as well as T cell-dependent antibody production and reduces viral replication and mutations	[30,83,92,154,157,158,159,160,161]
	- reduces platelets aggregation and consequent thrombosis	[98,162]
Magnesium	- reduces SARS-CoV-2 mortality	[31,32,34]
	- reduces:	
	(1) low grade persistent inflammation	[31,163]
	(2) endothelial dysfunction	[31,163]
	(3) thrombosis risk	[163]
	(4) cytokine storm	[164]
	(5) collagen deposition and tissue fibrosis	[165]
	- if used in patients with COVID-19, reduces the need of oxygen therapy and intensive care unit admission	[46]
	- reduces the risk of cardiac arrhythmias	[166]
Flavonoids and polyphenols: quercetin, resveratrol, sulforaphane	- anti-inflammatory activity through activation of Nrf2 pathway and stimulated production of anti-inflammatory enzymes and proteins	[116]
	- anti-inflammatory activity through inhibition of NF-κB signaling, resulting in down-regulation of proinflammatory cytokine production	[116]
	- antiviral effect by conformational isomerism and molecular docking with the enzymes used by virus for replication	[118,119,121,167,168,169,170]
	- reduces the risk of thrombosis by anti-platelets aggregation activity	[123,124,170,171,172]
	- reduces the risk of cerebrovascular pathology	[125]
	- reduces the risk of pulmonary fibrosis by inhibiting leukocyte proteases	[143]
Curcumin	- reduces symptoms, inflammatory responses, and mortality in patients with COVID-19	[127,128,173]
	- reduces platelets activation and coagulation throughout multiple pathways	[172,174]
Sulforaphane	- probably responsible for death rate reduction for COVID-19	[25,131]
	- improve Nrf2 signal, mitochondrial ATP synthesis, improves glucose and fat metabolism, anti-inflammatory effect through JNK/AP-1/NF-κB inhibition and Nrf2/HO-1 activation	[132]
	- reduces the risk of thrombosis by anti-platelets aggregation activity	[123]

## Data Availability

Not applicable.
